# Using image‐text similarity scores from picture descriptions for differentiating the logopenic variant of progressive aphasia from amnestic Alzheimer’s dementia

**DOI:** 10.1002/alz.094350

**Published:** 2025-01-09

**Authors:** Lokesha Pugalenthi, Heather R Dial, J. Jessy Li, Suzanne Schmitz, Maya L. Henry, Lisa Wauters, Jeffrey N. Keller, Avery Largent, Patrick Chang, Joshua Chang, Heather Foil, Rosemary A Lester‐Smith, Paul Toprac, Robin C. Hilsabeck

**Affiliations:** ^1^ Rice University, Houston, TX USA; ^2^ University of Houston, Houston, TX USA; ^3^ The University of Texas at Austin, Austin, TX USA; ^4^ Dell Medical School at The University of Texas at Austin, Austin, TX USA; ^5^ University of Texas, Austin, TX USA; ^6^ Pennington Biomedical Research Center, Baton Rouge, LA USA; ^7^ UT Health San Antonio, San Antonio, TX USA

## Abstract

**Background:**

Primary progressive aphasia (PPA) is a language‐led dementia associated with underlying Alzheimer’s disease (AD) or frontotemporal lobar degeneration pathology. As part of the Alzheimer’s spectrum, logopenic (lv) PPA may be particularly difficult to distinguish from amnestic AD, due to overlapping clinical features. Analysis of linguistic and acoustic variables derived from connected speech has shown promise as a diagnostic tool for differentiating dementia subtypes. Here we investigate the utility of a machine learning (ML)‐based classifier for differentiating lvPPA from amnestic AD using an image‐text similarity metric derived from a picture description task.

**Method:**

Monolingual English speakers diagnosed with lvPPA (n = 18) and amnestic AD (n = 14) were asked to describe a picture (Cat Rescue). A similarity score between the image and patient’s transcription was derived with each model of the Vision‐Language encoder CLIP. This involved computing the cosine similarity between the image‐ and transcription‐based representation vectors. These image‐text similarity scores were fed into 10 ML classification algorithms to differentiate lvPPA from amnestic AD.

**Result:**

For differentiating lvPPA from amnestic AD, our CLIP‐based feedforward neural network classifier achieved F1, AUC, and Accuracy scores of 81%, 80%, and 81% respectively. This outperformed our baselines of stratified random sampling and uniform random sampling by 30%.

**Conclusion:**

The current study shows promising differentiation of lvPPA from amnestic AD through the single feature of image‐text similarity. This approach holds potential as an automated connected speech analysis tool for differential diagnosis of dementia subtypes. Future directions include evaluating performance in a larger dataset and comparing the classification performance between image‐text similarity and acoustic‐linguistic features.

